# High spatial resolution artificial vision inferred from the spiking output of retinal ganglion cells stimulated by optogenetic and electrical means

**DOI:** 10.3389/fncel.2022.1033738

**Published:** 2022-12-09

**Authors:** Andreea Elena Cojocaru, Andrea Corna, Miriam Reh, Günther Zeck

**Affiliations:** ^1^Institute of Biomedical Electronics, TU Wien, Vienna, Austria; ^2^Institute for Ophthalmic Research at the University of Tübingen, Tübingen, Germany

**Keywords:** retinal ganglion cell, optogenetics, epiretinal electrical stimulation, channelrhodopsin-2, CMOS-based microelectrode array

## Abstract

With vision impairment affecting millions of people world-wide, various strategies aiming at vision restoration are being undertaken. Thanks to decades of extensive research, electrical stimulation approaches to vision restoration began to undergo clinical trials. Quite recently, another technique employing optogenetic therapy emerged as a possible alternative. Both artificial vision restoration strategies reported poor spatial resolution so far. In this article, we compared the spatial resolution inferred *ex vivo* under ideal conditions using a computational model analysis of the retinal ganglion cell (RGC) spiking activity. The RGC spiking was stimulated in epiretinal configuration by either optogenetic or electrical means. RGCs activity was recorded from the *ex vivo* retina of transgenic late-stage photoreceptor-degenerated mice (rd10) using a high-density Complementary Metal Oxide Semiconductor (CMOS) based microelectrode array. The majority of retinal samples were stimulated by both, optogenetic and electrical stimuli using a spatial grating stimulus. A population-level analysis of the spiking activity of identified RGCs was performed and the spatial resolution achieved through electrical and optogenetic photo-stimulation was inferred using a support vector machine classifier. The best f_1_ score of the classifier for the electrical stimulation in epiretinal configuration was 86% for 32 micron wide gratings and increased to 100% for 128 microns. For optogenetically activated cells, we obtained high f_1_ scores of 82% for 10 microns grid width for a photo-stimulation frequency of 2.5 Hz and 73% for a photo-stimulation frequency of 10 Hz. A subsequent analysis, considering only the RGCs modulated in both electrical and optogenetic stimulation protocols revealed no significant difference in the prediction accuracy between the two stimulation modalities. The results presented here indicate that a high spatial resolution can be achieved for electrical or optogenetic artificial stimulation using the activated retinal ganglion cell output.

## Introduction

Optogenetic therapy emerged as a method in modern neuroscience at the beginning of the 2000s, when several research groups showed the use of the technique for the light-induced control of the excitable cells’ activity (Bi et al., 2006; Ivanova et al., 2010). As of last year, we witnessed the results of the first patient in the clinical trial NCT03326336 to report a partial recovery of vision after undergoing optogenetic therapy (Sahel et al., 2021). Various approaches have been taken in the search for the most suitable cell type target for photo-stimulation: focusing on the bipolar cells (BC; Lagali et al., 2008; Doroudchi et al., 2011; Cehajic-Kapetanovic et al., 2015; Macé et al., 2015; van Wyk et al., 2015; Kralik et al., 2022; Reh et al., 2022), the remaining photoreceptors (Busskamp et al., 2010; Khabou et al., 2018) or the retinal ganglion cells (RGC; Jacobson et al., 2005; Bi et al., 2006; Ivanova et al., 2010; Gauvain et al., 2021; Reh et al., 2021). In the case of retinitis pigmentosa or age-related macular degeneration (AMD), the photoreceptor cell layers degrade progressively, leaving the RGC layer largely intact. In turn, this makes the RGCs a potentially attractive target. Recent evidence (Lu et al., 2020; Reh et al., 2022) compared the efficacy of targeting rodBCs or RGCs for optogenetic vision restoration and found that, despite the higher cell density and the ability of the BCs to preserve the inner information processing mechanism of the retina, RGCs are a potentially better target. This result is puzzling and may be attributed either to the strong spontaneous activity in the mouse line with targeted rod BCs (Reh et al., 2022) or to differences in the stimulation protocols.

In contrast, the activation of retinal cells through electrical stimulation has been studied for several decades now (Palanker and Goetz, 2018; Ayton et al., 2020), leading to market-approved electronic implants such as Argus II (Humayun et al., 2012; da Cruz et al., 2016; Second Sight, USA), Alpha AMS (Stingl et al., 2017; Zrenner et al., 2017; Palanker and Goetz, 2018; Ayton et al., 2020; Retina Implant, Germany) and IRIS II (Muqit et al., 2019; Pixium Vision, France). Implant prototypes include PRIMA (Palanker and Goetz, 2018; Palanker et al., 2020; Pixium Vision, France), which uses a subretinal configuration and infrared light stimulation on honeycomb structured photodiodes or POLYRETINA (Ferlauto et al., 2018; Chenais et al., 2021), aiming at a significant sight restoration. Despite the initial success, currently, there is no retinal prosthesis with CE or FDA approval on the market. One of the shortcomings of electrical stimulation is the relatively poor spatial resolution, which did not overcome legal blindness This had been attributed to either axonal activation for epiretinal stimulation (Beyeler et al., 2019, but see the alternative proposal in Weitz et al., 2015) or the spread of the stimulating unconfined electric field for subretinal stimulation (Palanker et al., 2005; Loudin et al., 2007; Wilke et al., 2011).

An open scientific question remained, if artificial stimulation applied directly to the retinal ganglion cells achieves a higher resolution as expected from the RGC’s morphology, which includes the soma but also the much larger dendritic tree and the elongated axons. Cell soma, dendrites, and axons are often transduced in optogenetic therapy (Shemesh et al., 2017; Forli et al., 2021; but see Greenberg et al., 2011) or may be activated by electrical stimulation (Beyeler et al., 2019; Tandon et al., 2021). To answer this question, we applied a stimulus protocol, where elongated grating patterns were reversed at a defined temporal frequency. This protocol has been previously reported for subretinal electrical (Lorach et al., 2015; Chenais et al., 2021) and for optogenetic stimulation (Cideciyan et al., 2016; Reh et al., 2021). The highest grating frequency which differentially activates RGCs determines the highest spatial resolution. Although previous reports from our lab (Corna et al., 2021; Reh et al., 2021) indicate that a high spatial resolution may be achieved by direct artificial stimulation of RGCs, the results were not conclusive for two reasons. Previous experiments were performed on different retinae, while the degree of retinal degeneration and remodeling had been reported to show a center-to-periphery gradient (Gargini et al., 2007). Secondly, the stimulation protocol used previously for electrical stimulation (Corna et al., 2021) was less suited to estimate spatial selectivity. To overcome these limitations we performed here artificial stimulations on the very same retinal portion in the same experimental condition and compared the inferred spatial resolution from the recorded RGC activity using the same computational analysis method.

The RGC neural activity was recorded using a Complementary Metal-Oxide-Semiconductor microelectrode array (CMOS-MEA) while stimulating the *ex vivo* retina of adult rd10 transgenic mice. We employed a support vector machine classifier to infer the spatial resolution achievable through either one of the two stimulation methods from the RGC spiking recorded in the same retinae to optogenetic and electrical stimuli.

## Materials and Methods

### Retinal preparation

*Ex vivo* retinae of adult, photoreceptor-degenerated *rd10* transgenic mice expressing the light sensitive opsin channel rhodopsin-2 (ChR2) in retinal neurons were used in this study. All experimental procedures were reported and approved by the Center for Biomedical Research, Medical University Vienna, Austria. In the current work, ChR2 was expressed in either rod-bipolar cells (*n* = 3 retinal samples, two female mice and one male, aged between 208 and 281 days post-natal) as described in Reh et al. (2022) or in the parvalbumin-positive retinal ganglion cells (*n* = 6 retinal samples from mice of either sex aged between 214 and 358 days post-natal) as described in Reh et al. (2021). At this age, all photoreceptors are fully degenerated (Chang et al., 2002; Gargini et al., 2007). Parvalbumin-positive RGCs represent approximately 30% of the total RGC population, comprising ON as well as OFF RGC classes (Yi et al., 2012). We could not identify ON or OFF RGC types here, since all photoreceptors degenerated at the age studies here (Cha et al., 2022).

**Figure 1:**
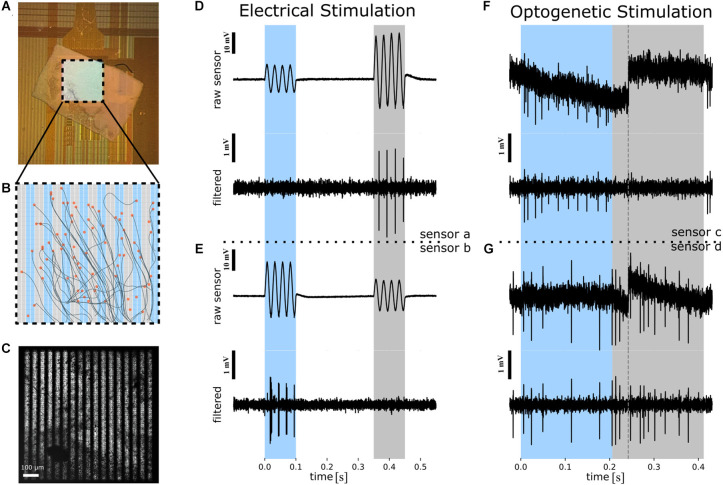
Overview of the experimental protocol used to infer the spatial resolution of the stimulus based on the RGC spiking. **(A)** Photograph of *ex vivo* rd10 retina interfaced to a CMOS-MEA. The central square (1 × 1 mm^2^) comprises 1,024 capacitive stimulation electrodes and 4,225 recording sites. **(B)** Schematic of the stimulation array with 1,024 stimulation electrodes. The recording sites are in the same area, with each stimulation electrode being enclosed by four recording sites (not shown here). Cell positions (orange circles) and inferred axon pathways are shown. In the background (blue and gray) the two phases of the reversed grating stimulus are shown. **(C)** Microscopic image of the retina on the CMOS MEA with the optogenetic photo-stimulation pattern superimposed. Scale bar: 100 μm. **(D)** The electrical stimulation protocol exemplified using the raw voltage of one recording sensor (recording site *a*). *Upper panel*: Four sinusoidal stimulation waveforms were applied to “blue” stimulation electrodes (panel **B**) between 0 and 100 ms, followed by four stimulation waveforms applied to the “gray” electrodes (panel **B**) between 350 and 450 ms. The selected recording sensor records a higher extracellular voltage artifact for stimulation with gray stimulation electrode as compared to stimulation with blue electrodes. Note that the same stimulus amplitude was applied to “blue” and “gray” electrodes. Scale bar: 10 mV. *Lower panel*: High-pass filtered extracellular voltage of the signal shown in the upper panel reveals spiking in each cycle of the four sinusoidal waveforms for stimulation with the gray electrodes only. Scale bar: 1 mV. **(E)** Detection of selective electrical activation revealed by a second sensor (“recording site b”). Description of the traces as in **(D)**. **(F)** The optogenetic stimulation protocol exemplified using the raw voltage of one recording sensor (recording site *c*). *Upper panel*: Spatially patterned photo-stimuli with fine gratings were projected onto selected regions of the CMOS sensor array (see panel **C**) between 0 and 200 ms, followed by a reverse grating between 200 and 400 ms. Dashed line in the raw extracellular voltage trace marks a sensor reset. Scale bar: 10 mV. *Lower panel*: High-pass filtered extracellular voltage of the signal shown in the upper panel reveals spiking during one of the stimulation phases (marked with “blue”). **(G)** Detection of selective optogenetic activation revealed by another sensor (“*recording site d*”). Description of the traces as in **(F)**. RGC, retinal ganglion cell; CMOS MEA, Complementary Metal-Oxide-Semiconductor microelectrode array.

The *ex vivo* retinal samples were prepared in carbogenated (95% O_2_, 5% CO_2_) Ames’ medium (Ames A1420, NaHCO_3_, Sigma Aldrich GmbH, Vienna, Austria), using dim red light under a dissection microscope (Leica Mikrosysteme G.m.b.H., Vienna, Austria). After mice were euthanized by cervical dislocation and after enucleation, a small incision was performed above the ora serrata to release the pressure inside the eye and ensure sample oxygenation. Next, a circular cut was made around the eyeball, the lens was removed and, by gentle movements of the forceps, the sclera and retinal pigment epithelium (RPE) were separated from the retina. Following the removal of the vitreous, a retinal portion was cut and placed on the sensor array of a CMOS-based MEA chip, with the RGC layer facing the recording sites (Figure 1A).

Prior to the sample preparation, the surface of the recording chip (CMOS MEA, see below) was cleaned with Tickopur (R60, 5%, 80°C, Dr. H. Stamm GmbH Chemische Fabrik, Berlin, Germany) and rinsed with distilled water. The active area was covered with a few microliters of poly-L-lysine (1 mg/ml, Sigma Aldrich GmbH, Vienna, Austria) in order to ensure tight tissue adhesion. The coating process proved to not affect the electrical stimulation, as shown in previous work (Eickenscheidt and Zeck, 2014; Stutzki et al., 2016).

The chip’s chamber, containing the retinal sample was filled with Ames’ medium and perfused constantly with the same carbonated solution to ensure the cells’ viability. The temperature in the recording chamber was kept between 34 and 36°C. All recordings were conducted in the dark.

### Electrical stimulation

In the current study, we used a commercial CMOS-MEA5000 system (Multi Channel Systems MCS GmbH, Reutlingen, Germany) for simultaneous stimulation and recording and CMOS-MEA chips as described previously (Bertotti et al., 2014; Corna et al., 2021). The chips comprise a recording array with 65 by 65 recording sites each of them separated by 16 μm and a second stimulation array comprising 32 by 32 stimulation sites with a center-to-center distance of 32 μm. The array of recording sites is interspersed with stimulation electrodes. To increase the stimulation strength (i.e., capacitive stimulation current), the top oxide was omitted leaving the chip surface with a native oxide only (Corna et al., 2021).

We applied cosine stimuli, as described in Corna et al. (2021), delivered at 40 Hz and a peak-to-peak amplitude of 2.5 V applied between the capacitive electrode and the ground electrode (Ag/AgCl) submerged in the recording solution. The selected stimulation charge density was 15 μC/cm^2^ and it was determined as described in Corna et al. (2018). The stimuli were presented as alternating gratings with a phase reversal. In the following, we will refer to the two spatial patterns per stimulation as “phase 1” and “phase 2”. The electrical stimuli were detected by the neighboring sensors as sinusoidal stimulus artifacts.

### Photo-stimulation

Intense light stimulation was delivered by a CoolLED pE-4000 system (CoolLED Ltd., Andover, UK) projecting onto a digital mirror device (Rapp OptoElectronic GmbH, Wedel, Germany). The patterned light stimuli (460 nm) were focused onto the retina through a microscope (Olympus) equipped with a 5× objective (Olympus MPlanFL N). This combination of systems allows for a patterned stimulation with micrometer range precision (Figure 1C). We projected the grating stimuli at two frequencies, 2.5 Hz, as previously used in Reh et al. (2022) and at 10 Hz (Reh et al., 2021).

### Data processing

Prior to the statistical analyses, the raw extracellular voltages recorded during optogenetic stimulation were filtered with a second order high pass Bessel filter at 200 Hz and a second order low pass Bessel filter at 5,000 Hz (Figure 1G), while the raw data from the electrical stimulation was high pass filtered with a fourth order Butterworth function at 1,000 Hz and low pass filtered with a second order Bessel function at 3,000 Hz (Figures 1D,E). Due to the sensor drift (Figure 1F) introduced by the blue light used for optogenetic stimulation, a sensor reset (duration 200 μs) was applied every 400 ms. This method, however, comes with the cost of introducing an additional artifact. In order to remove it, we used a method described in Reh et al. (2021) in which the raw data is divided in time intervals according to the times of the sensor reset. From these sequences of data, a “drift curve”, the result of a 2nd order Savitzky-Golay filter with an 800 μs windows, was subtracted from the raw data and this new dataset was filtered with a high-pass, 2nd order Butterworth filter of 100 Hz. Following this procedure, the signal data corresponding to the timestamps in which the sensor reset occurred was replaced by noise taken from the same recorded data. The last step in the preprocessing stage was further filtering the data, as described previously.

Spike sorting for the identification of single RGC units was performed using the CMOS-MEA-Tools software (Multi Channel Systems MCS GmbH, Reutlingen, Germany), which applies a sorting algorithm based on independent component analysis (Leibig et al., 2016).

A manual curation was applied to the sorted datasets to ensure that the cells were properly identified. The measures of interest for this post-processing step were IsoIBG, separability, and Signal-to-Noise-Ratio (SNR). IsoIBG (Neymotin et al., 2011) is a measure of the separation, in feature space, between the neural signal amplitudes and the other peak amplitudes, considered as background noise. When the sorter identifies only a single waveform cluster, the IsoIBG gets assigned a not a number value (NaN). Therefore, we chose to keep only the units (cells) which had numerical values. Separability measures the separation between different units’ clusters and the dimensions dominated by noise. A higher value of the separability ensures a clearer neural spike-noise distinction, hence the choice of imposing a 2.5 threshold on this metric. As the IsoIBG alone is not always a very informative metric (Neymotin et al., 2011), we considered also the SNR as being within the range of 3.3 and 14 for the case of electrical stimulation recordings.

For a qualitative estimation of how well RGCs are activated by artificial stimuli we calculated the relative change in firing rate as follows:


$$\text{RFR = }\frac{FR_{phase1}-FR_{phase2}}{FR_{phase1}+FR_{phase2}}$$

**Equation 1.** The formula for the relative change in firing rate (RFR) of the RGCs identified. *FR*_phase1_ and *FR*_phase2_ stand for firing rate values in phase 1 and firing rate values in phase 2, respectively.

### Pattern classification using support vector machines

The statistical analysis of the data was performed using a classification method, the support vector machines^1^ (Drucker et al., 1997; Pisner and Schnyer, 2019). Due to its relative ease on the resources during the training phase and its performance, it began to gain popularity in the bioengineering field (Pisner and Schnyer, 2019).

In brief, this method uses a way of finding a hyperplane in an n-dimensional space, where n is the number of features, that distinctly classifies data points. The hyperplanes are, in fact, the decision boundaries of the classifier and the data points falling on either side of the hyperplane will be assigned to one class or the other. To maximize the margin of the classifier, the algorithm makes use of the so-called “support vectors”, which are data points that are found closer to the hyperplane and influence its position and orientation. The main goal of the classifier is to minimize the errors. To do so, it uses a cost function to perform the learning and the optimizations; in this case, the cost function is the hinge loss.

In our current approach, we calculated the number of spikes per stimulus duration in each of the repetitions for every cell in a recording and constructed an input variable (x), a vector of a length n equal to the number of cells in a given recording. The total dataset thus constructed consisted of m such vectors, with m being the number of repetitions for a recording. Ultimately, the task was a binary classification problem of assigning the vector of firing rates in one repetition and phase to one of the two phases. Out of the classifier’s results, we could then infer the spatial resolution achieved by the stimulation protocols.

Specifically, for our case, we used the SVM () class of scikit-learn’s module, with a radial basis function kernel (rbf) and we imposed a regularization by a C constant of 1. The goal of such a kernel transformation is to project the original data point into a new dimensionality such that it becomes easier to separate the data belonging to each class using simple linear methods. Since scaling is a sensitive matter in the training of such a classifier, we investigated multiple scaling methods, concluding that the best performance for the given datasets is achieved by standardizing the values using the Standard Scaler () method, which follows a formula as in Eq. 2.


$$z=\frac{\left(x-\mu \right)}{\sigma }$$

**Equation 2.** The standard score formula as calculated for a sample x. μ denotes the mean of the training samples, while σ denotes the standard deviation of the training samples.

To ensure the reproducibility of our experiment, the randomness was controlled by setting a seed to a constant. The datasets were split into train and test sample sizes in a ratio of 80/20. The ability of the model to generalize was evaluated through cross-validation. We made use of the StratifiedKFold() class of scikit-learn, with a 10-fold split. Using this method, random parts of the dataset are selected and the results of the classifier’s performance are returned in the form of an average value.

One way to visualize the outcomes of the classification task is to use a confusion matrix (Tharwat, 2018), such as in Table 1. Different metrics typically used to assess the quality of the results, such as accuracy, precision, or recall are further extracted from this matrix.

**Table 1:** Confusion matrix table.

	Predicted	
Actual	True positive	False positive
	(TP)	(FP)
		Type I error
	False negative	True negative
	(FN)	(TN)
	Type II error	

The values computed are typically given in percentages and correspond to the percentage of either correctly or wrongly classified data points. The confusion matrix is used to calculate the f_1_ score (Eq. 3).

To evaluate the classifier’s performance, we referred to the f_1_ score, a metric which can be calculated as the harmonic mean of the precision and recall, as in Eq. 3.


$$f_{1}=2\frac{P\;\cdot \;R}{P+R}\equiv \frac{TP}{TP+\frac{1}{2}(FP+FN)}$$

**Equation 3.** f_1_ score formula. Here, P denotes the value of the precision and R the value of the recall, respectively. TP refers to the number of true positives, FP the value of the false positives, and FN the value of the false negatives. For the definition of TP, FP, and FN see Table 1.

## Results

In this study, we investigated the spatial resolution achieved through electrical or optogenetic stimulation of RGCs in adult transgenic rod degenerated mice (rd10-ChR2). The retina was interfaced with the RGC layer facing the recording and stimulation sites of CMOS MEA chips (Figure 1A). This high density of the recording sites allows electrical imaging of somatic and axonal activity in a large RGC population (Figure 1B) as well as the option to electrically stimulate the retinal neurons. Within the 10 different retinal portions stimulated and analyzed in the following we identified artificial activation in over 800 RGCs.

### Patterned photo-stimulation of optogenetically transduced retinal neurons with fine gratings evokes spatially confined RGC activation

In the first experiment we investigated the response of RGCs upon photo-stimulation of optogenetically transduced retinal neurons. Patterned photo-stimulation *in vitro* was applied at high precision using a focused stimulus projection through a microscope objective (Figure 1C). We, therefore, investigated protocols using alternating gratings with a bar width ranging between 5 and 128 μm. The RGC spiking activity during stimulus presentation was recorded and assigned to individual RGCs (see “Materials and methods” section, Figure 1B).

**Figure 2:**
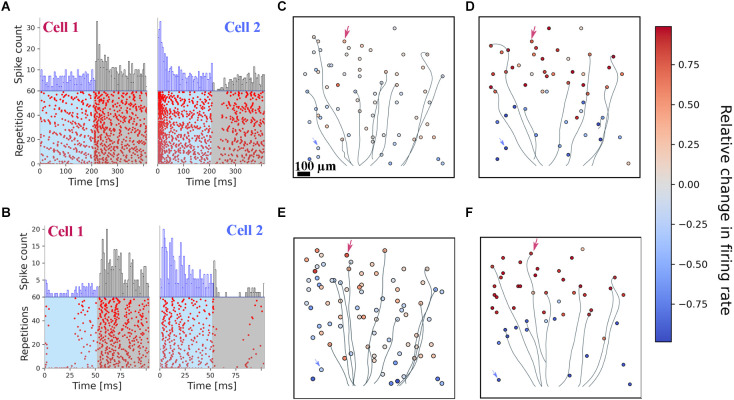
Optogenetic stimulation with fine gratings evokes spatially restricted RGC spiking. All results presented here originate from one retina. **(A)** Raster plots of two exemplary RGCs stimulated for 24 s with an alternating stimulus at a spatial frequency of 50 μm. Every 200 ms, the spatial pattern was switched. A preferential activation is detected for both cells. **(B)** Raster plots of the very same RGCs, for a stimulus presented at 10 Hz temporal switching and a 50 μm grating width. Both RGCs cells show a preferential response to one of the two stimulation phases. **(C,D)** Spatial mapping of identified RGCs for the 2.5 Hz pattern switching with a 50 μm **(C)** and 500 μm **(D)** grating width. Color coded is the relative change in firing rate between the two stimulation phases. Arrows indicate the two selected cells’ positions depicted in panel **(A)**. RGC axons are inferred from the spike-triggered averaging; only a few of them are shown here for visualization purposes. **(E,F)** Spatial mapping of identified RGCs for the 10 Hz pattern reversal with a spatial frequency of 50 μm **(E)** and 500 μm **(F)**. Color coded is the relative change in firing rate between the two stimulation phases. Arrows indicate the two selected cells’ positions depicted in panel **(B)**.

As a qualitative presentation of the stimulated cells’ spiking activity, raster plots of selected RGCs obtained after repetitive stimulation with grating pattern reversals of one spatial frequency are shown in Figures 2A,B. The raster plots show the spiking activity recorded from two cells to a spatial grating comprising 50 μm wide bars and reversed with a frequency of either 2.5 Hz or 10 Hz. For both frequencies, a robust spiking was recorded without any sign of fading, i.e., decrease of the spiking with an increasing repetition rate. The peri-stimulus histograms in Figures 2A,B qualitatively show the preference of each of the two selected cells for one grating phase. This result is surprising, given that the dendritic tree of RGCs spans about 200 μm in diameter and thus each phase of the grating covers part of the dendrites. In Supplementary Figure 1 we present the raster plots and PSTH of two RGCs to optogenetic stimulation with very fine gratings (10 μm).

To quantify the activation of RGCs by photo-stimulation we calculated the relative change in firing rate (RFR, defined in the “Materials and methods” section) for the two phases of the grating. An RFR value of 1 implies activity exclusively in phase 1, a value of −1 exclusive activation in phase 2. The RFR for the cell population stimulated with the 50 and 500 μm grating is shown in Figures 2C,D (for 2.5 Hz), and Figures 2E,F (for 10 Hz), respectively. Electrical imaging of the interfaced retina allows identification of the RGC position and for most cells of the corresponding axon. Axons of a few selected RGCs are included here as well. Although the photostimulus was presented across the axon in each phase, we did not detect axonal activation. This is most obvious for large gratings (500 μm; Figures 2D,F), where the stimulus can be easily detected by the eye from the relative change in firing rate for both frequencies (2.5 Hz and 10 Hz). For this example, phase 1 stimulated the upper part of the RGCs on the array.

We investigated gratings ranging between 5 and 100 μm (with 500 μm serving as a control). The distribution of RFR values obtained for the individual grid width is shown in Supplementary Figure 2. We note that for each grating the distribution is different from a unimodal distribution (Hartigan dip test). These RFRs different from zero suggests that a computational model, which uses a large RGC population (>30 cells) and thereby “considers” cells with non-zero RFRs may succeed to infer the correct stimulus (i.e., grating pattern). In the next step we analyzed the effect of electrical stimulation and return to the discrimination task using a computational model.

### Electrical stimulation with fine gratings evokes spatially confined RGC activation

We next analyzed the RGC spiking upon electrical stimulation using a grating stimulus (Figure 1B) analogous to the optogenetic photostimulation. The grating comprised a set of electrodes arranged in rectangular shapes (1 × 1 mm^2^) with a grid width of either 32, 64 or 128 μm (see “Materials and methods” Section). During one stimulus period, which we referred to as “phase 1” (100 ms long), every second column (32 × 1 electrodes) stimulated the retina while for the alternating columns the electrode voltage was left floating. After a break of 250 ms, the electrodes in the spatial “phase 2” were stimulated. The spatial resolution of the grating stimulus is limited by the size of stimulation electrode (32 μm).

**Figure 3:**
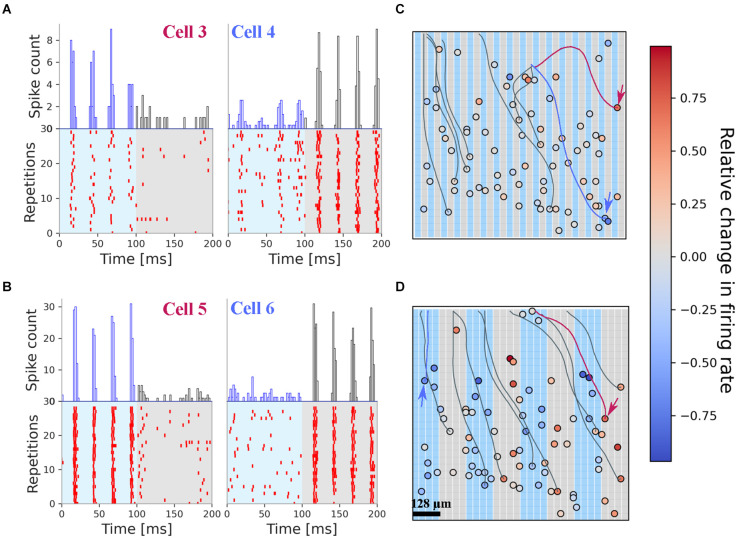
Electrical stimulation with fine gratings evokes spatially restricted RGC spiking. All results presented here originate from one retina. **(A)** Rasterplot of two selected RGCs upon stimulation with fine grating stimuli (bar width: 32 μm). Cell 3 is stimulated only in phase 1 (0–100 ms) while cell 4 is stimulated in phase 2 (100–200 ms). The cell positions are marked with arrows in panel **(C)**. **(B)** Rasterplot of two selected RGCs upon stimulation with grating stimuli (bar width: 128 μm). The spiking pattern for each cell is confined to one phase. The cell positions are marked with arrows in panel **(D)**. **(C)** Positions of the RGC on the stimulation array. Color-coded is the relative change in firing rate. The background colors of the grating mark the electrodes used in phase 1 and phase 2. The results presented here were obtained after stimulation with 32 μm narrow gratings (panel **A**). For visualization purposes, only a few axons are shown. **(D)** Positions of the RGC on the stimulation array, with the corresponding color coding of the relative change in firing rate. Note the dominant red colors on top of the gray stimulation electrodes indicating selective activation by this stimulus.

The spiking activity of two exemplary RGCs to 30 repetitions of the same stimulus pattern (32 μm grid width) is shown in Figure 3A. The spiking in the interstimulus interval is not shown. The two cells were selected to demonstrate their selective activation by one single phase but not the second. Note, that the two cells are activated within each phase of the four sinusoidal stimuli without fading (i.e., decrease in firing rate). A second example (Figure 3B) from the same retina shows an even stronger firing and selective activation of the RGC in only one of the grating phases, i.e., grating reversals. The stronger response is probably caused by the extended stimulation area, now covering 4 × 32 single electrodes (i.e., 128 × 1,024 μm^2^). For these RGCs, no weak activation in the non-preferred phase was detected.

The relative change in firing rate (RFR) of 80 RGCs in one retina sample for the 32 microns grating stimulus is shown in Figure 3C, while the RFR of these RGCs responding to the 128 microns grating stimulus is shown in Figure 3D. We note, that most RGCs are activated by both stimuli—however, for the narrow gratings the number dropped slightly. No effort was undertaken here to clarify the difference in identified RGCs. There are 45% of the RGCs where the RFR is higher than 0.01. Thus, as a first qualitative result, we report that electrical stimuli with spatial gratings as small as 32 μm evoke different spiking activity in the stimulated RGCs. This qualitative result was found in all investigated retinae. In a control experiment, we investigated an rd10 retina, which did not express ChR2. Here again, we found the same high discrimination result.

In addition to the RGC soma our algorithm identifies the corresponding axon. Exemplary axons are shown in Figures 3C,D. We note that these axons cross the stimulation electrodes and may potentially be activated. However, the selective stimulation shown in Figures 3A,B for four RGCs demonstrate that axons are not activated here. The avoidance of axonal stimulation in epiretinal configuration for low-frequency (40 Hz) stimuli strengthens the result of previous reports, where the stimulus shape was a small square (Corna et al., 2021) or a single circular electrode (Weitz et al., 2015).

### Discrimination of fine spatial grating stimuli using computational modeling of the stimulated RGC spiking

In the next step we employed a support vector machine (SVM) classifier to infer the presented stimulus (“phase 1” or “phase 2”) from the firing rates of the RGCs. Given the values of the firing rate of the recorded cells in each of the stimulation phases in a training set, the classifier was asked to assign the correct stimulation phase for a test set. The training comprised 80% of the stimulus repetitions, while the remaining repetitions were used to test the discrimination performance.

**Figure 4:**
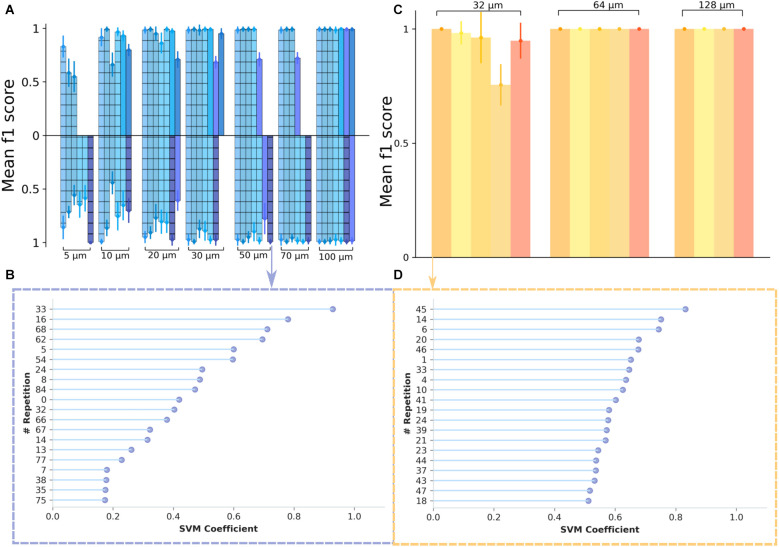
Inferring the spatial resolution of optogenetically and electrically stimulated RGCs from a support vector machines classifier. **(A)** Classification results are shown as f_1_ score values for data obtained from optogenetic experiments. The different colors denote different experimental days. On the upper side of the plot are data from the 2.5 Hz switching frequency and on the lower side are data from the 10 Hz switching frequency. A classifier’s performance is considered to be significant when the f_1_ score is above the “chance level” which, in this case, we considered to be at 0.5. With the exception of one recording day, the metrics show a notably high value for all of the spatial frequencies considered, reaching saturation at around 30 μm for 2.5 Hz and 50 μm for 10 Hz. **(C)** Classification results for data obtained from electrical stimulation experiments. The different colors correspond to different experimental days. The saturation is reached at 64 μm for all datasets considered. However, for some experimental days, we note particularly high values for the 32 μm grating width as well. **(B,D)** Feature importance plot showing the most significant 20 features (vectors containing firing rate values for individual stimulus repetitions) for the classifier. In panel **(B)** there is an exemplary plot for good classification results, for the case of the 50 μm grating width, with a 10 Hz pattern reversal from an optogenetic stimulation dataset, while in panel **(D)** the plot shows an example of the feature importance for the 32 μm grating width, in the case of an electrical stimulation dataset.

We chose to refer to the f_1_ score as a metric and, to test the robustness of the model, we used a 10 fold cross-validation (see “Materials and methods” Section), while also shuffling the data to ensure a random selection. In the first analysis, we investigated the cell responses to different switching frequencies: at 10 Hz and 2.5 Hz, respectively. The predictions (f_1_-score) for optogenetic stimulation and various spatial frequencies are shown in Figure 4A (frequency 2.5 Hz—upper bar plots; 10 Hz—lower bar plots. The results are presented separately for recordings made on retinae where ChR2 was expressed in RGCs (hashed bars) and for recordings where ChR2 was expressed in rod BCs (filled bars). We obtained remarkably high metrics in the case of optogenetic stimulation. The results indicate that the classifier performs well, with only small standard deviation values, ranging between 1% and 12%, and f_1_ scores above 73% for all experiments with gratings at least 10 μm wide. Individual values varied between preparations. Without differentiating between the two strains, the average f_1_-score for the grating of 30 μm was 93.7% for 2.5 Hz (*n* = 8 experiments), 94.3% for 10 Hz (*n* = 6 experiments), 83% for the grating of 10 μm at 2.5 Hz frequency switch (*n* = 6 experiments), and 73.1% for 10 Hz (*n* = 6 experiments). Considering the difference between the strains we noted, for the 20 μm grating and a 2.5 Hz stimulation a score of 95.5% for the samples where ChR2 was expressed in the RGCs (*n* = 5 experiments) and 84.1% for the other mouse strain (*n* = 2 experiments), while for 30 μm, delivered at 2.5 Hz, a score of 98.3% was obtained for samples with transduced RGCs (*n* = 5 experiments) and 87.48% for the samples with transduced rod BCs (*n* = 3 experiments). Although the number of recordings prevents a rigorous test for statistical significance, we identify a high prediction accuracy for narrow gratings in both mouse strains.

The high prediction accuracy is quite remarkable, provided that both gratings with narrow stripes stimulate the RGCs. We asked, if the high prediction value is mainly determined by one or a few stimulated RGCs. The percentage of contributing cells was above 20% of the whole population irrespective of the stimulation frequencies (2.5 and 10 Hz).

These values are in agreement with a previous study on optogenetic stimulation of RGCs (Reh et al., 2021). However, we also had recordings where the classifier showed poor performance. To identify a potential source, we plotted the most informative features, the ones with the highest coefficients of the SVM, considering data from optogenetic stimulation with a phase reversal at 10 Hz and a spatial frequency of 50 μm (Figure 4B). From Figure 4B; we see that the coefficients are not uniformly distributed, the classifier assigns weights to the incorrectly classified data.

Next, we asked how well the two grating stimuli are discriminated upon electrical epiretinal stimulation. After initial processing of the recordings, we employed the support vector machines (SVM) classifier to infer the stimulation phase from the firing rates of the RGC. The firing rates of all identified RGCs (irrespective if they were stimulated or not) were used as input values of the SVM (see “Materials and methods” Section).

To test the robustness of the model, we used the StratifiedKFold() method, with 10 folds, while also shuffling the data to ensure a random selection. The results (Figures 4C,D) indicate that the classifier performs well, with only small standard deviation values, ranging between 5% and 13%. The mean f_1_-score for electrical stimulation with a 32 μm wide grating stimulus was 86.4% (*n* = 5 experiments) and higher for larger gratings.

**Figure 5:**
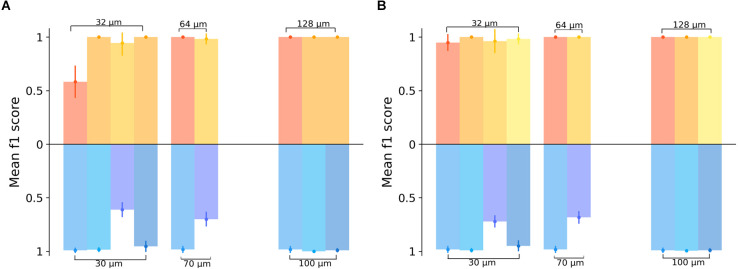
Comparing the spatial resolution of electrically or optogenetically stimulated RGCs in the same retinal sample. **(A)** Classification results of the grating pattern reversal stimuli inferred from a subset of RGCs responding to both stimulation types, electrical (yellow) and optogenetic (blue). In the upper part of the graph, the yellow color-coded bars show the results obtained from four different electrically stimulated retinae. The blue scheme shows the results for the same responsive RGCs in the same four retinae upon optogenetic stimulation. Each bar for the electrical stimulation corresponds to a bar on the optogenetic stimulation at the same x-position on the graph, indicating that the same experimental day and the same sample was used in the analysis. We note that for gratings wider 32 μm not all protocols were applied or activated sufficient RGCs. **(B)** Classification results for the full datasets in the four retinae stimulated by electrical and optogenetic means, i.e., with RGCs responding to either electrical or optogenetic stimulation. In the upper part of the graph, the yellow color-coded bars show the results from the RGC datasets upon electrical stimulation, while in the lower part the prediction results inferred from the optogenetic stimulation are shown. Each bar for the electrical stimulation corresponds to a bar on the optogenetic stimulation at the same x-position on the graph, indicating that the same sample was considered in both cases.

Finally, we evaluated four recordings were for both, optogenetic and electrical stimulation a sufficiently large number of retinal ganglion cells (>30 RGCs) was responsive to both stimulation modalities. Recordings where either modality activated too few cells were not compared as this biased the evaluation. We first restricted the evaluation to the subset of RGCs activated by electrical and optogenetic stimuli (Figure 5A). The average prediction accuracy (f_1_ score) based on this subset was: 87.7% for electrical stimulation with 32 μm gratings and 89.8% for optogenetic stimulation with 30 μm gratings. These average values are not statistically different (Wilcoxon-Rank-Sum test); however, the low number of retinal samples compared here (*n* = 4) prevents a rigorous interpretation. The number of RGCs identified upon electrical or optogenetic stimulation in one single retina is not identical. We, therefore, compared the prediction for the four retina using for each retinae the entire responsive RGC population (Figure 5B). The prediction results increased only slightly for both electrical and optogenetic stimulation when considering the full RGC population activated by each modality (average f_1_ score = 97.8% for electrical stimuli with 32 μm gratings vs. average f_1_ score = 90.1% for optogenetic stimulation with 30 μm gratings). On average, high discrimination f_1_ scores were detected irrespective of the stimulation method, when considering retinae stimulated by both modalities.

## Discussion

In this study, we estimated the spatial resolution achieved by electrical or optogenetic artificial stimulation using the spiking output of the activated retinal ganglion cells. The artificial stimuli were applied in epiretinal configuration to late-stage photoreceptor degenerated retina. The spatial resolution was inferred from the discrimination of grating pattern reversal stimuli based on a computational model using the activity of retinal ganglion cells. The model provided a high degree of discriminability for optogenetic stimuli with very fine gratings (f_1_ score > 73%, 10 μm bar width) and a similarly high degree of discrimination for electrical stimuli (>86%, 32 μm bar width). These results are remarkable given that the retinal ganglion cell’s morphology extends over a much larger diameter.

In the following we relate our results to previous work, discuss them from a biophysical perspective and conclude with suggestions how these results may be used for the development of further retinal implants.

The results presented here aimed to clarify the question, how the two investigated artificial stimulation strategies (optogenetic or electrical) compare when applied to the *ex vivo* retina. To eliminate variability across preparations we investigated the very same *ex vivo* retina subjected to both stimulus modalities, which had not been the case in previous studies (Corna et al., 2021; Reh et al., 2021, 2022). Our results indicate that high and very similar discrimination scores are obtained for the two stimulus modalities using the same computational model. The discriminability of electrical grating stimuli (32 μm–0.54 cpd) and of optogenetic stimuli (10 μm–1.75 cpd) can only be explained by a local stimulation of cell somata and/or axon initial segment while avoiding dendrites and distal axonal compartments. The stimulation of the axon initial segment, the most excitable cellular compartment (Fried et al., 2009; Werginz et al., 2014) cannot be identified or distinguished in this study from the stimulation of the cell body.

For optogenetic stimulation we observed two spiking patterns in the stimulated RGCs: if ChR2 was expressed in rod bipolar cells, we detected RGC spiking always in both phases of the grating stimulus. This result indicates qualitatively that even at the late retinal degeneration stage the synaptic connectivity between bipolar cells and retinal ganglion cells is preserved. In contrast, when ChR2 was expressed in PV-positive RGCs we detected often increased spiking in one of the two stimulus phases only (Figure 2A). This result indicates that for optogenetic stimulation at either 10 or 2.5 Hz only the cell soma and/or the axon initial segment is activated. The results for optogenetic stimulation extend those presented earlier by our lab (Reh et al., 2021, 2022). For stimulation of optogenetically transduced rod bipolar cells, the discrimination reported previously (Reh et al., 2022) was not as high as reported here. Possible reasons might be the different computational algorithm as well as variability in the recordings.

The estimation of spatial resolution for electrical stimuli followed the approach used by Lorach et al. (2015) and by Chenais et al. (2021) and is spatially equivalent to the optogenetic stimulus. However, in contrast to, the pulsatile stimuli used in previous work (Lorach et al., 2015) we did not encounter fading of the induced RGC spiking activity, but a reliable response (Figures 3A,B) to all sinusoidal stimuli. More interestingly, we did not detect spiking activity caused by the grating stimulus for one of the reversals. This is in contrast to the results obtained for short pulsatile waveforms (Lorach et al., 2015; Chenais et al., 2021) and may help to solve the open question of avoidance of axonal stimulation (Beyeler et al., 2019; Tandon et al., 2021; Vilkhu et al., 2021) in epiretinal configuration. The grating stimulus used here isolates the source of activation, i.e., helps separate between activation of the soma and distal axons. If distal axons would be activated the RGC spike pattern should be identical for every stimulus phase. Our results of phase-specific RGC activation (Figures 3A,B) indicate direct somatic stimulation. For narrow grids (bar width: 32 μm), we detected for some cells a stimulation in both stimulus phases. This suggests that for the monopolar configuration used here the electric field is not confined to the 32 μm above the electrodes but spreads laterally. A better discrimination and refinement may be obtained if the return electrode is close to the stimulation electrode (Ho et al., 2018) or by three-dimensional structures (Flores et al., 2019). Note, that for grids with a high spatial resolution we detected few cells with a relative firing rate exceeding 0.1. Therefore, the statement of high spatial resolution in artificial vision should be carefully distinguished from the statement of identifying small objects, which had been addressed i.e., by Chenais et al. (2021). The latter appears more difficult and potentially conflicting with electrode safety issues (Corna et al., 2018).

In laboratory conditions, we and others have reported high spatial and temporal resolution of stimulated RGC activity both for epiretinal electrical (Weitz et al., 2015; Corna et al., 2021) and optogenetic stimulation (Ferrari et al., 2020; Gauvain et al., 2021; Reh et al., 2021; Chaffiol et al., 2022; Khabou et al., 2022). This represents an encouraging result for future vision restoration. However, it also contrasts with many *in vivo* studies (Tomita et al., 2010; Ganjawala et al., 2019; McGregor et al., 2020), including the reports from human patients (Humayun et al., 2012; Stingl et al., 2017; Palanker et al., 2020; Sahel et al., 2021). We speculate that in laboratory conditions precise optical focus is easily achieved but may represent a challenge for most *in vivo* optogenetic conditions (Ronzitti et al., 2017). Similarly, for *ex vivo* electrical stimulation the interfacing between stimulation electrodes and RGCs within the recording time of a few hours is very tight (Zeitler et al., 2011) in contrast to the ~20μm assumed *in vivo* (Ayton et al., 2020). Furthermore, the poor resolution achieved by electrical epiretinal implants had been attributed to axonal activation leading to elongated percepts (Beyeler et al., 2019). Sinusoidal stimuli likely improve the resolution (Weitz et al., 2015; Corna et al., 2021), but this had not been tested in clinical trials. Finally, we have implicitly assumed here that the differential response of RGCs to different artificial grating stimuli can be transferred to visual acuity. This needs to be confirmed in future studies using healthy retina and physiological light stimuli.

One of the future goals may therefore be to consider the lessons learned in recent years for future implants. Several promising developments are underway, including the optimization of electrical stimulation waveforms (Schütz et al., 2020; Löhler et al., 2022), optimization of optogenetic transduction using novel AAV viruses (Gauvain et al., 2021), and focused photo-stimulation (Ronzitti et al., 2017; Emiliani et al., 2022).

## Data Availability Statement

The datasets presented in this study can be found in online repositories. The names of the repository/repositories and accession number(s) can be found below: https://gitlab.com/CAE157/frontiers_in_neuroscience_code_base.

## Author Contributions

ACoj performed the data analysis, part of the experiments, and wrote the manuscript. ACor and MR gathered experimental data. GZ contributed to the experimental planning and manuscript writing. All authors contributed to the article and approved the submitted version.

## Footnotes

^1^Support Vector Machines. https://scikit-learn.org/stable/modules/svm.html

## References

[B1] Ayton L. N. Barnes N. Dagnelie G. Fujikado T. Goetz G. Hornig R.. (2020). An update on retinal prostheses. Clin. Neurophysiol. 131, 1383–1398. 10.1016/j.clinph.2019.11.029 31866339 PMC7198351

[B2] Bertotti G. Barnes N. Dagnelie G. Fujikado T. Goetz G. Hornig R.. (2014). “A CMOS-based sensor array for in-vitro neural tissue interfacing with 4225 recording sites and 1024 stimulation sites,” in IEEE 2014 Biomedical Circuits and Systems Conference, BioCAS 2014—Proceedings, (Lausanne, Switzerland: Institute of Electrical and Electronics Engineers Inc.), 131, 304–307. 10.1109/BioCAS.2014.6981723

[B3] Beyeler M. Nanduri D. Weiland J. D. Rokem A. Boynton G. M. Fine I. (2019). A model of ganglion axon pathways accounts for percepts elicited by retinal implants. Sci. Rep. 9:9199. 10.1038/s41598-019-45416-4 31235711 PMC6591412

[B4] Bi A. Cui J. Ma Y.-P. Olshevskaya E. Pu M. Dizhoor A. M.. (2006). Ectopic expression of a microbial-type rhodopsin restores visual responses in mice with photoreceptor degeneration. Neuron 50, 23–33. 10.1016/j.neuron.2006.02.026 16600853 PMC1459045

[B5] Busskamp V. Duebel J. Balya D. Fradot M. Viney T. J. Siegert S.. (2010). Genetic reactivation of cone photoreceptors restores visual responses in retinitis pigmentosa. Science 329, 413–417. 10.1126/science.1190897 20576849

[B6] Cehajic-Kapetanovic J. Eleftheriou C. Allen A. E. Milosavljevic N. Pienaar A. Bedford R.. (2015). Restoration of vision with ectopic expression of human rod opsin. Curr. Biol. 25, 2111–2122. 10.1016/j.cub.2015.07.029 26234216 PMC4540256

[B7] Cha S. Ahn J. Jeong Y. Lee Y. H. Kim H. K. Lee D.. (2022). Stage-dependent changes of visual function and electrical response of the retina in the rd10 mouse model. Front. Cell. Neurosci. 16:926096. 10.3389/fncel.2022.926096 35936494 PMC9345760

[B8] Chaffiol A. Provansal M. Joffrois C. Blaize K. Labernede G. Goulet R.. (2022). *In vivo* optogenetic stimulation of the primate retina activates the visual cortex after long-term transduction. Mol. Ther. Methods Clin. Dev. 24, 1–10. 10.1016/j.omtm.2021.11.009 34977267 PMC8671818

[B9] Chang B. Hawes N. L. Hurd R. E. Davisson M. T. Nusinowitz S. Heckenlively J. R. (2002). Retinal degeneration mutants in the mouse. Vis. Res. 42, 517–525. 10.1016/s0042-6989(01)00146-8 11853768

[B10] Chenais N. A. L. Airaghi Leccardi M. J. I. Ghezzi D. (2021). Photovoltaic retinal prosthesis restores high-resolution responses to single-pixel stimulation in blind retinas. Commun. Mater. 2:28. 10.1038/s43246-021-00133-2

[B11] Cideciyan A. V. Roman A. J. Jacobson S. G. Yan B. Pascolini M. Charng J.. (2016). Developing an outcome measure with high luminance for optogenetics treatment of severe retinal degenerations and for gene therapy of cone diseases. Invest. Ophthalmol. Vis. Sci. 57, 3211–3221. 10.1167/iovs.16-19586 27309625 PMC4928698

[B13] Corna A. Herrmann T. Zeck G. (2018). Electrode-size dependent thresholds in subretinal neuroprosthetic stimulation. J. Neural Eng. 15:045003. 10.1088/1741-2552/aac1c8 29717707

[B12] Corna A. Ramesh P. Jetter F. Lee M.-J. Macke J. H. Zeck G. (2021). Discrimination of simple objects decoded from the output of retinal ganglion cells upon sinusoidal electrical stimulation. J. Neural Eng. 18:46086. 10.1088/1741-2552/ac0679 34049288

[B14] da Cruz L. Dorn J. D. Humayun M. S. Dagnelie G. Handa J. Barale P. O.. (2016). Five-year safety and performance results from the argus II retinal prosthesis system clinical trial. Ophthalmology 123, 2248–2254. 10.1016/j.ophtha.2016.06.049 27453256 PMC5035591

[B15] Doroudchi M. M. Greenberg K. P. Liu J. Silka K. A. Boyden E. S. Lockridge J. A.. (2011). Virally delivered channelrhodopsin-2 safely and effectively restores visual function in multiple mouse models of blindness. Mol. Ther. 19, 1220–1229. 10.1038/mt.2011.69 21505421 PMC3129568

[B16] Drucker H. Greenberg K. P. Liu J. Silka K. A. Boyden E. S. Lockridge J. A.. (1997). Support vector regression machines. Adv. Neural Inform. Process. Sys. 19, 155–161.

[B17] Eickenscheidt M. Zeck G. (2014). Action potentials in retinal ganglion cells are initiated at the site of maximal curvature of the extracellular potential. J. Neural Eng. 11:036006. 10.1088/1741-2560/11/3/036006 24762943

[B18] Emiliani V. Entcheva E. Hedrich R. Hegemann P. Lüscher C. Mahn M.. (2022). Optogenetics for light control of biological systems. Nat. Rev. Methods Primers 2:55. 10.1038/s43586-022-00136-4 PMC10627578 37933248

[B19] Ferlauto L. Leccardi M. J. I. A. Chenais N. A. L. Gilliéron S. C. A. Vagni P. Bevilacqua M.. (2018). Design and validation of a foldable and photovoltaic wide-field epiretinal prosthesis. Nat. Commun. 9:992. 10.1038/s41467-018-03386-7 29520006 PMC5843635

[B20] Ferrari U. Deny S. Sengupta A. Caplette R. Trapani F. Sahel J. A.. (2020). Towards optogenetic vision restoration with high resolution. PLoS Comput. Biol. 16:e1007857. 10.1371/journal.pcbi.1007857 32667921 PMC7416966

[B21] Flores T. Huang T. Bhuckory M. Ho E. Chen Z. Dalal R.. (2019). Honeycomb-shaped electro-neural interface enables cellular-scale pixels in subretinal prosthesis. Sci. Rep. 9:10657. 10.1038/s41598-019-47082-y 31337815 PMC6650428

[B22] Forli A. Pisoni M. Printz Y. Yizhar O. Fellin T. (2021). Optogenetic strategies for high-efficiency all-optical interrogation using blue-light-sensitive opsins. eLife 10:e63359. 10.7554/eLife.63359 34032211 PMC8177884

[B23] Fried S. I. Lasker A. C. W. Desai N. J. Eddington D. K. Rizzo J. F. 3rd (2009). Axonal sodium-channel bands shape the response to electric stimulation in retinal ganglion cells. J. Neurophysiol. 101, 1972–1987. 10.1152/jn.91081.2008 19193771 PMC4588392

[B24] Ganjawala T. H. Lu Q. Fenner G. W. Abrams T. H. Pan Z.-H. (2019). Improved CoChR variants restore visual acuity and contrast sensitivity in a mouse model of blindness under ambient light conditions. Mol. Ther. 27, 1195–1205. 10.1016/j.ymthe.2019.04.002 31010741 PMC6554551

[B25] Gargini C. Terzibasi E. Mazzoni F. Strettoi E. (2007). Retinal organization in the retinal degeneration 10 (rd10) mutant mouse: a morphological and ERG study. J. Comp. Neurol 500, 222–238. 10.1002/cne.21144 17111372 PMC2590657

[B26] Gauvain G. Akolkar H. Chaffiol A. Arcizet F. Khoei M. A. Desrosiers M.. (2021). Optogenetic therapy: high spatiotemporal resolution and pattern discrimination compatible with vision restoration in non-human primates. Commun. Biol. 4:125. 10.1038/s42003-020-01594-w 33504896 PMC7840970

[B27] Greenberg K. P. Pham A. Werblin F. S. (2011). Differential targeting of optical neuromodulators to ganglion cell soma and dendrites allows dynamic control of center-surround antagonism. Neuron 69, 713–720. 10.1016/j.neuron.2011.01.024 21338881

[B28] Ho E. Smith R. Goetz G. Lei X. Galambos L. Kamins T. I.. (2018). Spatiotemporal characteristics of retinal response to network-mediated photovoltaic stimulation. J. Neurophysiol. 119, 389–400. 10.1152/jn.00872.2016 29046428 PMC5867391

[B29] Humayun M. S. Dorn J. D. Dagnelie G. Sahel J. A. Stanga P. E. (2012). Interim results from the international trial of second sight’s visual prosthesis. Ophthalmology 119, 779–788. 10.1016/j.ophtha.2011.09.028 22244176 PMC3319859

[B30] Ivanova E. Hwang G. S. Pan Z.-H. Troilo D. (2010). Evaluation of AAV-mediated expression of Chop2-GFP in the marmoset retina. Invest. Ophthalmol. Visual Sci. 51, 5288–5296. 10.1167/iovs.10-5389 20484599 PMC2939198

[B31] Jacobson S. G. Aleman T. S. Cideciyan A. V. Sumaroka A. Schwartz S. B. Windsor E. A.. (2005). Identifying photoreceptors in blind eyes caused by *RPE65* mutations: prerequisite for human gene therapy success. Proc. Natl. Acad. Sci. U S A 102, 6177–6182. 10.1073/pnas.0500646102 15837919 PMC1087926

[B32] Khabou H. Garita-Hernandez M. Chaffiol A. Reichman S. Jaillard C. Brazhnikova E.. (2018). Noninvasive gene delivery to foveal cones for vision restoration. JCI Insight 3:e96029. 10.1172/jci.insight.96029 29367457 PMC5821199

[B33] Khabou H. Garita-Hernandez M. Chaffiol A. Reichman S. Jaillard C. Brazhnikova E.. (2022). Optogenetic targeting of AII amacrine cells restores retinal computations performed by the inner retina. bioRxiv [Preprint]. 10.1101/2022.07.28.501925 PMC10589896 37868206

[B34] Kralik J. Stocker N. Kleinlogel S. (2022). Bipolar cell targeted optogenetic gene therapy restores parallel retinal signaling and high-level vision in the degenerated retina. Commun. Biol. 5:116. 10.1038/s42003-022-04016-1 36266533 PMC9585040

[B35] Lagali P. S. Balya D. Awatramani G. B. Münch T. A. Kim D. S. Busskamp V.. (2008). Light-activated channels targeted to ON bipolar cells restore visual function in retinal degeneration. Nat. Neurosci. 11, 667–675. 10.1038/nn.2117 18432197

[B36] Leibig C. Wachtler T. Zeck G. (2016). Unsupervised neural spike sorting for high-density microelectrode arrays with convolutive independent component analysis. J. Neurosci. Methods 271, 1–13. 10.1016/j.jneumeth.2016.06.006 27317497

[B37] Löhler P. Pickhinke A. Erbslöh A. Kokozinski R. Seidl K. (2022). “SoC for retinal ganglion cell stimulation with integrated sinusoidal kilohertz frequency waveform generation,” in 2022 17th Conference on Ph.D Research in Microelectronics and Electronics (PRIME), (Villasimius, SU, Italy: IEEE), 271, 341–344. 10.1109/PRIME55000.2022.9816766

[B38] Lorach H. Goetz G. Smith R. Lei X. Mandel Y. Kamins T.. (2015). Photovoltaic restoration of sight with high visual acuity. Nat. Med. 21, 476–482. 10.1038/nm.3851 25915832 PMC4601644

[B39] Loudin J. D. Simanovskii D. M. Vijayraghavan K. Sramek C. K. Butterwick A. F. Huie P.. (2007). Optoelectronic retinal prosthesis: system design and performance. J. Neural Eng. 4, S72–S84. 10.1088/1741-2560/4/1/S09 17325419

[B40] Lu Q. Ganjawala T. H. Krstevski A. Abrams G. W. Pan Z. H. (2020). Comparison of AAV-mediated optogenetic vision restoration between retinal ganglion cell expression and ON bipolar cell targeting. Mol. Ther. Methods Clin. Dev. 18, 15–23. 10.1016/j.omtm.2020.05.009 32548211 PMC7287188

[B41] Macé E. Lu Q. Ganjawala T. H. Krstevski A. Abrams G. W. Pan Z. H.. (2015). Targeting channelrhodopsin-2 to ON-bipolar cells with vitreally administered AAV restores ON and OFF visual responses in blind mice. Mol. Ther. 23, 7–16. 10.1038/mt.2014.154 25095892 PMC4270733

[B42] McGregor J. E. Godat T. Dhakal K. R. Parkins K. Strazzeri J. M. Bateman B. A.. (2020). Optogenetic restoration of retinal ganglion cell activity in the living primate. Nat. Commun. 11:1703. 10.1038/s41467-020-15317-6 32245977 PMC7125151

[B43] Muqit M. M. K. Velikay-Parel M. Weber M. Dupeyron G. Audemard D. Corcostegui B.. (2019). Six-month safety and efficacy of the intelligent retinal implant system II device in retinitis pigmentosa. Ophthalmology 126, 637–639. 10.1016/j.ophtha.2018.11.010 30591229

[B44] Neymotin A. S. Lytton W. W. Olypher A. V. Fenton A. A. (2011). Measuring the quality of neuronal identification in ensemble recordings. J. Neurosci. 31, 16398–16409. 10.1523/JNEUROSCI.4053-11.2011 22072690 PMC3247202

[B45] Palanker D. Goetz G. (2018). Restoring sight with retinal prostheses. Physics Today 71, 26–32. 10.1063/PT.3.3970 31885403 PMC6934168

[B46] Palanker D. Vankov A. Huie P. (2005). Design of a high-resolution optoelectronic retinal prosthesis. J. Neural Eng. 2, S105–S120. 10.1088/1741-2560/2/1/012 15876646

[B47] Palanker D. Le Mer Y. Mohand-Said S. Muqit M. Sahel S. A. (2020). Photovoltaic restoration of central vision in atrophic age-related macular degeneration. Ophthalmology 127, 1097–1104. 10.1016/j.ophtha.2020.02.024 32249038 PMC7384969

[B48] Pisner D. A. Schnyer D. M. (2019). “Support vector machine,” in Machine Learning: Methods and Applications to Brain Disorders, 31, (Cambridge, Massachusetts, USA: Academic Press), 101–121. 10.1016/B978-0-12-815739-8.00006-7

[B49] Reh M. Lee M. J. Schmierer J. Zeck G. (2021). Spatial and temporal resolution of optogenetically recovered vision in ChR2-transduced mouse retina. J. Neural Eng. 18:056013. 10.1088/1741-2552/abe39a 33545694

[B50] Reh M. Lee M.-J. Zeck G. (2022). Expression of channelrhodopsin-2 in rod bipolar cells restores ON and OFF responses at high spatial resolution in blind mouse retina. Adv. Ther. 5:2100164. 10.1002/adtp.202100164

[B51] Ronzitti E. Ventalon C. Canepari M. Forget B. C. Papagiakoumou E. Emiliani V. (2017). Recent advances in patterned photostimulation for optogenetics. J. Opt. 19:113001. 10.1088/2040-8986/aa8299

[B52] Sahel J.-A. Boulanger-Scemama E. Pagot C. Arleo A. Galluppi F. Martel J. N.. (2021). Partial recovery of visual function in a blind patient after optogenetic therapy. Nat. Med. 27, 1223–1229. 10.1038/s41591-021-01351-4 34031601

[B53] Schütz H. Boulanger-Scemama E. Pagot C. Arleo A. Galluppi F. Martel J. N.. (2020). “Pseudo-resistor based attenuator as an efficient electrode driver for sinusoidal stimulation of retinas,” in Proceedings - IEEE International Symposium on Circuits and Systems, (Seville, Spain: Institute of Electrical and Electronics Engineers Inc.), 1–5. 10.1109/ISCAS45731.2020.9180425

[B54] Shemesh O. A. Tanese D. Zampini V. Linghu C. Piatkevich K. Ronzitti E.. (2017). Temporally precise single-cell-resolution optogenetics. Nat. Neurosci. 20, 1796–1806. 10.1038/s41593-017-0018-8 29184208 PMC5726564

[B55] Stingl K. Schippert R. Bartz-Schmidt K. U. Besch D. Cottriall C. L. Edwards T. L.. (2017). Interim results of a multicenter trial with the new electronic subretinal implant alpha AMS in 15 patients blind from inherited retinal degenerations. Front. Neurosci. 11:445. 10.3389/fnins.2017.00445 28878616 PMC5572402

[B56] Stutzki H. Helmhold F. Eickenscheidt M. Zeck G. (2016). Subretinal electrical stimulation reveals intact network activity in the blind mouse retina. J. Neurophysiol. 116, 1684–1693. 10.1152/jn.01095.2015 27486110 PMC5144714

[B57] Tandon P. Bhaskhar N. Shah N. Madugula S. Grosberg L. Fan V. H.. (2021). Automatic identification of axon bundle activation for epiretinal prosthesis. IEEE Trans. Neural Syst. Rehabil. Eng. 29, 2496–2502. 10.1109/TNSRE.2021.3128486 34784278 PMC8860174

[B58] Tharwat A. (2018). Classification assessment methods. Appl. Comput. Informatics 17, 168–192. 10.1016/j.aci.2018.08.003

[B59] Tomita H. Sugano E. Isago H. Hiroi T. Wang Z. Ohta E.. (2010). Channelrhodopsin-2 gene transduced into retinal ganglion cells restores functional vision in genetically blind rats. Exp. Eye Res. 90, 429–436. 10.1016/j.exer.2009.12.006 20036655

[B60] van Wyk M. Pielecka-Fortuna J. Löwel S. Kleinlogel S. (2015). Restoring the ON switch in blind retinas: opto-mGluR6, a next-generation, cell-tailored optogenetic tool. PLoS Biol. 13:e1002143. 10.1371/journal.pbio.1002143 25950461 PMC4423780

[B61] Vilkhu R. S. Madugula S. S. Grosberg L. E. Gogliettino A. R. Hottowy P. Dabrowski W.. (2021). Spatially patterned bi-electrode epiretinal stimulation for axon avoidance at cellular resolution. bioRxiv [Preprint]. 10.1088/1741-2552/ac3450 PMC8736333 34710857

[B62] Weitz A. C. Nanduri D. Behrend M. R. Gonzalez-Calle A. Greenberg R. J. Humayun M. S.. (2015). Improving the spatial resolution of epiretinal implants by increasing stimulus pulse duration. Sci. Transl. Med. 7:318ra203. 10.1126/scitranslmed.aac4877 26676610 PMC4698804

[B63] Werginz P. Fried S. I. Rattay F. (2014). Influence of the sodium channel band on retinal ganglion cell excitation during electric stimulation - a modeling study. Neuroscience 266, 162–177. 10.1016/j.neuroscience.2014.01.067 24560986 PMC4423397

[B64] Wilke R. G. H. Moghadam G. K. Lovell N. H. Suaning G. J. Dokos S. (2011). Electric crosstalk impairs spatial resolution of multi-electrode arrays in retinal implants. J. Neural Eng. 8:046016. 10.1088/1741-2560/8/4/046016 21673395

[B65] Yi C.-W. Yu S.-H. Lee E.-S. Lee J.-G. Jeon C.-J. (2012). Types of parvalbumin-containing retinotectal ganglion cells in mouse. Acta Histochem. Cytochem. 45, 201–210. 10.1267/ahc.11061 22829714 PMC3394870

[B500] Zeitler R. Fromherz P. Zeck G. (2011). Extracellular voltage noise probes the interface between retina and silicon chip. Appl. Phys. Lett. 99:263702. 10.1063/1.367222422829714

[B66] Zrenner E. Yi C. W. Yu S. H. Lee E. S. Lee J. G. Jeon C. J.. (2017). “The subretinal implant ALPHA: implantation and functional results,” in Artificial Vision, eds Gabel V. (Cham: Springer), 65–83. 10.1007/978-3-319-41876-6_6

