# Supplementary material for: High spatial resolution artificial vision inferred from the spiking output of retinal ganglion cells stimulated by optogenetic and electrical means
Source: Front Cell Neurosci. 2022 Dec 9;16:1033738. doi: 10.3389/fncel.2022.1033738 (PMC9780279; doi:10.3389/fncel.2022.1033738)
Supplement: Supplementary file 1 [file Data_Sheet_1.PDF]

Supplementary Material

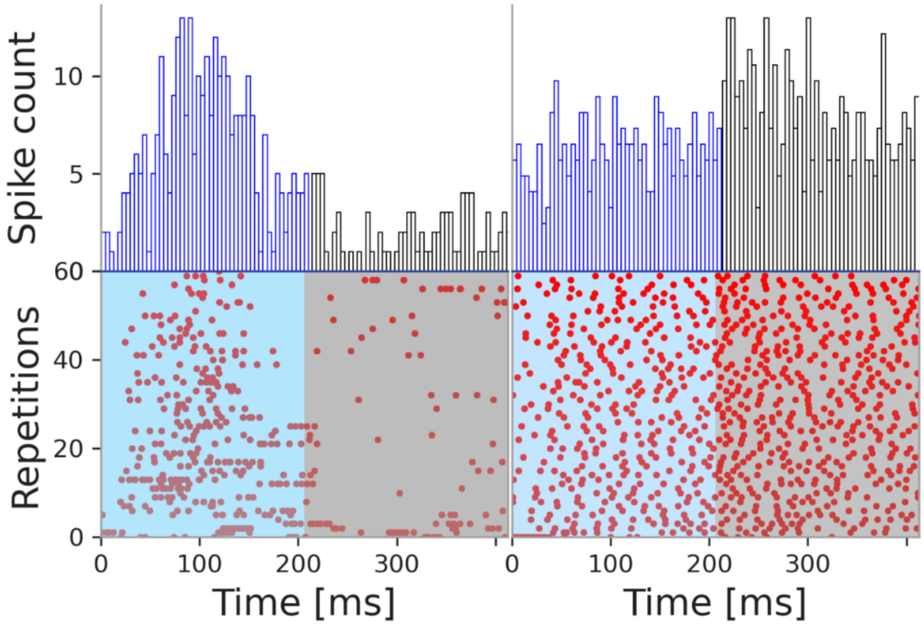

Supplementary Figure 1.

Optogenetic stimulation with fine gratings (10 µm width) evokes spatially selective RGC spiking. The raster plots and corresponding peri-stimulus time histograms originate from two different RGC in two different retinae.

Left panel: Spike data obtained upon 60 repetitions of the grating reversal stimulus from an RGC in a retina where ChR2 was expressed in the rod bipolar cells.
Right panel: Spike data obtained upon 60 repetitions of the grating reversal stimulus from an RGC in a retina where ChR2 was expressed in the parvalbumin-positive ganglion cells.
Both stimulations protocols were performed at identical reversal frequency (2.5 Hz).

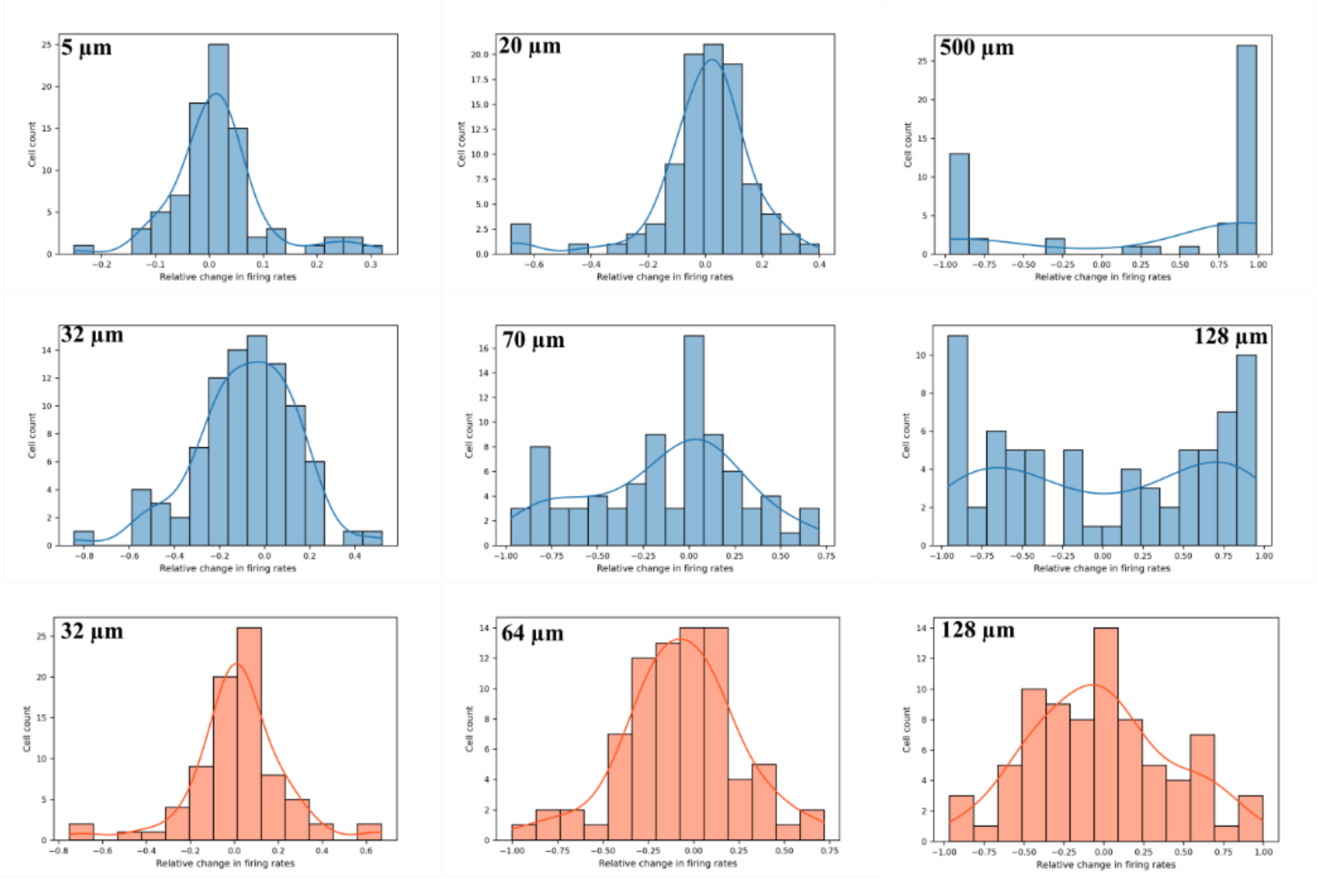

Supplementary Figure 2.

Relative change in firing rate (RFR) distributions of selected RGC populations upon optogenetic (**blue**) or electrical (**orange**) stimulation. Each subplot was generated upon stimulation with a different grating (i.e. first subplot with 5 µm wide grating). The multimodal distributions indicate a preference of the cells to either one of the stimulation phases. For a grid width of 20 µm (optogenetic stimulus) or 32 µm (electrical or optogenetic stimulus) we identified several cells where the relative firing rate RFR changes by more than 10%. For large grid widths (e.g. 500 µm or 128 µm), the RFR distributions upon optogenetic stimulation are bimodal.
